# Criminal Abortion in America

**Published:** 1860-03

**Authors:** 


On Criminal Abortion in America. By Horatio R. Storer, M. D., Member of the American Medical Association. Philadelphia: J. B. Lippincott & Co. 1860.

Although criminal abortion is practised to a fearful extent, we hope for the sake of humanity, as we verily believe, that Dr. Storer makes out too strong a case in the above named pamphlet. We have reason to know that the *morale* of criminal abortion is of the darkest character. That it is a crime equal in heinousness to that of murder, every physiologist knows. Hence no excuse should be allowed to palliate the punishment of it, as there is certainly none for its perpetration. The most alarming and the least excusable feature of its practice is, as we think, derived from the character and standing of the criminals. We believe from our own experience, as well as the testimony of our professional *confreres*, that the vast majority of the perpetrators of this fiendish infanticide, are people in what are styled the better classes of society; that many of them are not only held in high esteem by the world, but are active members of christian churches. Nay, Ministers of the Gospel, of spotless reputation before the congregations and communities in which they reside and officiate; these clergymen and church-members often try to suborn the consciences of the members of our profession; oftner they employ the means furnished by cormorant-quacks and professional abortionists. Parents engaged in this practice imbue their hands in the blood of their own children; either directly, by immediate interference, or indirectly, by winking at its perpetration in their own families. They will tell any body who remonstrates with them that they do not view the matter in the light of a crime against the law of man, or a crime against God's law. Now we believe this assertion as reluctantly as we credit the statements of those unfortunate married men with whom we often meet, who contract gonorrhœa or syphilis from the seats of unclean privies. However unfortunate and damnable the practice in this class of people, and the encouragement their example affords to the commission in detail of this great sin, their degradation places them upon the highest round of the ladder of human depravity, compared to the depth in which the wholesale dealers in human blood are sunk, who are spreading all over the land, the information and means necessary to enable and tempt many who would otherwise be innocent of a thought in that direction, to strangle their own offspring. I allude to the proprietors of newspapers, who for money publish and recommend; and the druggists who so unblushingly placard and sell abortive nostrums and instruments. The manufacturers of these nostrums, and professional abortionists, are fiends comparable in their turpitude to nothing outside the gates of the infernal regions.

The above pamphlet is calculated to do good, and we are sorry that the people themselves cannot have the same facts set before them, as widely as the charlatan advertisements, which do so much to promote the evil this monograph is intended to expose. Let the public read it, and it will blush with shame at the extent and horrible bearing it has upon all the sacred ties and bonds which link mankind together.

